# Research on the paths and strategies of the integrated development of culture and tourism industry in urban historical blocks

**DOI:** 10.3389/fpubh.2022.1016801

**Published:** 2022-11-07

**Authors:** Feng Liang, Yu Pan, Meilong Gu, Yamei Liu, Lei Lei

**Affiliations:** ^1^Tourism Research Centre, Wuxi Institute of Technology, Wuxi, China; ^2^College of Business, Wuxi Taihu University, Wuxi, China; ^3^College of Business, Shanghai University of Finance and Economics, Shanghai, China; ^4^School of Humanities, Jiangnan University, Wuxi, China

**Keywords:** Qingming Bridge, historic and cultural blocks, integrated development of culture and tourism, cultural heritage, theoretical framework

## Abstract

COVID-19 has brought about great impact on the global economy. Various countries have adopted different levels of spatial isolation measures to curb the spread of the epidemic. These measures not only limit the spatial flow of people and property, but also cause global anxiety and public mental health problems. Corresponding to this process, cultural demands are growing stronger and the humanistic shift in today's urban economic development also puts forward higher requirements for local culture. Historic districts are an important symbolic historical and cultural landscape of a city, and many cities regard them as important starting points for the shaping of urban characteristics and competitiveness. Taking Qingming Bridge Historical and Cultural Block in Wuxi City as an example, combined with the public's mental health needs in the context of COVID-19 and the current background of the return of humanism in urban development, and construct a more sustainable framework for the renewal and development of urban historical blocks, focusing on the integration and development of the cultural landscape and tourism in the historical block. A comprehensive analysis shows that the development of Qingming Bridge Historical and Cultural Block must be based on the comprehensive investment of “four types of resources,” such as culture, land, capital, and labor, and must rely on the cooperation of the “two sectors” of culture and tourism. By earnestly respecting the above, as well as the “triple bottom line” of regional ecology, economy, and society, sustainable development can be achieved.

## Introduction

The COVID-19 pandemic has caused unprecedented disruption to the global economic system, and nearly all countries have taken corresponding measures to curb the rapid spread of COVID-19, including social distancing, space lock-downs, and curfews. It greatly restricts the international, inter-regional and intra-regional free flow of human, financial and other elements, thereby accelerating the collapse of tourism and related industries. Not only that, according to the World Health Organization, the COVID-19 pandemic has increased the global prevalence of anxiety and depression by 25%, so it is recommended that all countries must pay more attention to the field of mental health and better support the mental health of their people. At present, under the background of the global victory over the epidemic, many cities are seeking comprehensive recovery and development after the epidemic. How to develop healthier and more sustainable development has become a topic of great concern to every city.

Every city is actually an organic form of life (1). In the context of globalization, a city can greatly improve its comprehensive competitiveness by shaping its local characteristics and actively showing its local history and geographic image to the outside world (2). For modern cities, historical blocks are an important place for human beings to live and move together, an important space in which to express their cultural evolution, vivid evidence that constitutes the memory of historical cities, and a part of human daily life. Many cities take this as an important starting point to shape urban characteristics and competitiveness (3). However, urban competition is also a “double-edged sword,” which can not only become the main driving force of urban development under the guidance of urban administrative power, but can also cause imbalance and chaos within regional development, which seriously restricts urban development. As an important social practice activity in the market economy, urban competition requires epistemological and methodological innovations on the basis of inheriting the essence of traditional thought.

With the development of urbanization, urban land resources have changed from processes of “incremental expansion” to “stock optimization,” and the regeneration and reproduction of old city space has become an inevitable demand (4). As the spatial imprint of the process of interaction between man and nature, and between man and man, the cultural relics and historic sites in the historical blocks are relatively concentrated distributed, which can well reflect the traditional style and local characteristics of a certain historical period, and often become an important historical symbol and cultural landscape of a city (5). Since this cultural landscape has important social value, it is often internalized in various social and economic activities, thereby making urban economic development more sustainable (6, 7). To revive historical districts, many cities have vigorously opened up tourism and implemented various cultural activities (8). Tourism drives the physical space expansion of historical blocks, greatly enriches the tourist consumption space of blocks, and at the same time, increases the possibility of space consumption in historical and cultural blocks, thereby driving urban development (9, 10).

**Figure 1:**
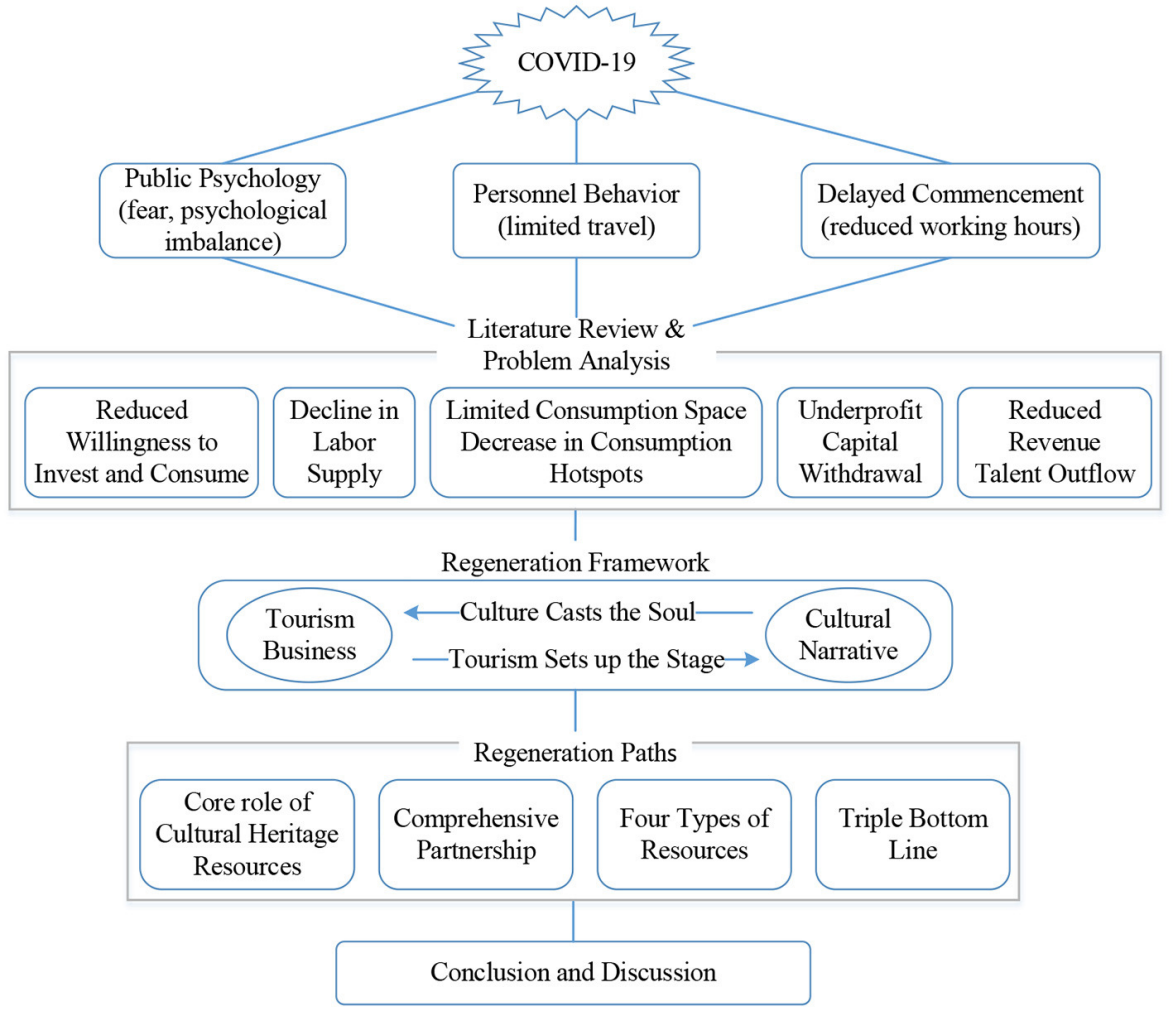
The logical thinking of the research.

This article largely takes the Qingming Bridge Historical and Cultural Block in Wuxi City as a case study, fully considers the background of the overall impact of COVID-19 and the troubled urban economic development (Figure 1), and builds a sustainable development framework for the integration of culture and tourism in the urban historical and cultural block. It breaks the inertial development thinking in the past process of urban renewal and development, which focused too much on economic or material elements, and often ignored non-material elements. The Qingming Bridge Historical and Cultural Block is the location of the world cultural heritage. Its paths and strategies for urban development and renewal, multi-business integration, especially the integration of culture and tourism, can also provide ideas and models for other urban blocks. It is universal and can enable people to deal with the integrated development of urban cultural landscape and tourism after the epidemic in a more rational and sustainable perspective, thereby further promoting the healthy development of historical blocks and realizing the grand vision of “better city, better life.”

## Literature review

By combing the research literature on historical blocks since the 20th century, it can be seen that people have a gradually developed cognitive process regarding the importance of historical blocks in urban development, and their understanding of the objects to be protected in historical blocks has developed as follows: “single architecture–historic districts–neighborhoods and environment–historic districts in urban development.” France was one of the first countries in the world to call for the protection of cultural heritage. Its “Historical Cultural Relics Law” promulgated in 1913 requires the state to protect the buildings that are historically or artistically in the public interest. It is the promulgation of this benchmark law and the subsequent establishment of a series of related regulations that have changed people's perception of cultural heritage and prompted people to truly regard cultural heritage as the public wealth of mankind (11). The Athens Charter adopted in August 1933, first called for buildings and neighborhoods with historical value to be properly preserved and not destroyed (12). People's awareness of the integrity and historicity of historical relics began with the 1964 Venice Charter, and the 1976 Nairobi Recommendations further clarified and accelerated the protection of historical towns and neighborhoods, but the Venice Charter was not passed until 1987. The Charter for the Preservation of Historic Towns and Urban Areas (also known as the Washington Charter) formally defines a historic district as: “Historic urban areas, large and small, including cities, towns and historic centers or quarters, together with their natural and man-made environments. Beyond their role as historical documents, these areas embody the values of traditional urban cultures” (13).

The research on the protection, renewal, and development of historical blocks has made much progress in moving from theory to practice. From simply the physical space, the focus has gradually shifted to humanistic elements and sustainable urban economic development. The core research topics include five research dimensions from the outside to the inside, including the theory of neighborhood protection and renewal, appearance renovation, social issues, public participation, and tourism development. Among these dimensions, in terms of the research on conservation and renewal theory, after experiencing large-scale reconstruction in the 18th century, the decay of old cities in the 19th century, and the adjustment of renewal methods, the renewal theories since the 20th century have paid more attention to the idea of sustainable development and are more targeted and able to solve practical problems. In terms of the renovation of historical districts, both the theoretical and practical aspects demonstrate that urban planning and visible material improvement must be carried out simultaneously, not only to expand and improve the functions of residences and the environment, but also to retain the traditional district culture to bring vitality to the district. In terms of research on social issues in blocks, this should not only focus on people-oriented planning at the planning level, but should also start to project planning into the practice of the spatial layout of blocks, paying more attention to social space fairness, improvement to the residents' quality of life, and the revival of block vitality. After the introduction of “housing leading” and “industry and Commerce leading” concepts, the “tourism leading” viewpoint appeared, and it was verified with numerous cases that the development of modern tourism can bring about further revival of historic districts (14–16).

“Culture is the soul of tourism, and tourism is the carrier of culture” (17, 18); thus, the integration of culture and tourism has now formed a relatively mature research paradigm (19, 20). The integration of culture and tourism has an important impact on the urban process in at least two aspects (21, 22). On the one hand, sorting out the regional cultural context has endowed physical space with more humanistic connotations, strengthened the unique attributes of the consumption space, and improved its consumption value (23, 24). On the other hand, the constant flow of tourist visits has expanded the recognition and influence of regional cultural resources, which has helped to exert the extensive linkage effect of cultural and tourism resources (25, 26). There is also a very important practical reason for the integration of culture and tourism, that is, in the context of the huge challenges brought by COVID-19 to global development, especially the spatial isolation of people, the psychological impact is the most profound. The implantation of cultural soul elements in the development process of the material economy is an important source of making up for the public's psychological needs, and it can provide a lasting driving force for social and economic development.

## Study area

The development of some famous canal cities in the world, such as the Suez Canal, the Panama Canal, the Pontequisist Waterway Bridge and Canal, etc., has formed their own characteristics. Some cities along the route attach great importance to the protection of the ecological authenticity of the canal itself, such as the Rideau Canal, Suez Canal, Panama Canal, Canal Midi, etc.; Some cities condense the experience and wisdom of people's canal management by law, such as the Erie Canal in the northeastern United States, which incorporates the protection, display, utilization of canal heritage and other works of canal governance into the rule of law system; Other cities, on the other hand, attach great importance to the integrated development of the canal cultural tourism industry, and promote the deep integration and development of the canal cultural tourism industry by rationally planning the canal cultural route, promoting the overall publicity of the canal culture, and cultivating a unified and recognizable canal cultural tourism brand, such as Amsterdam Canal in the Netherlands, Otaru Canal in Japan, etc. Qingming Bridge Historical and Cultural District is situated in the Wuxi section of the Beijing-Hangzhou Grand Canal, which is located in the economically developed circle of the Yangtze River Delta, and the integration of culture and tourism is urgent and typical in this region. Since 2018, the system reform and industrial practice of China's cultural and tourism integration have accelerated in this region, and the cultural and tourism industry that “fits to be integrated and can be fully integrated” has begun to form stronger business relationships and market radiation (27), therefore the study of Qingming Bridge Historical and Cultural District has a strong representative.

### Brief introduction

**Figure 2:**
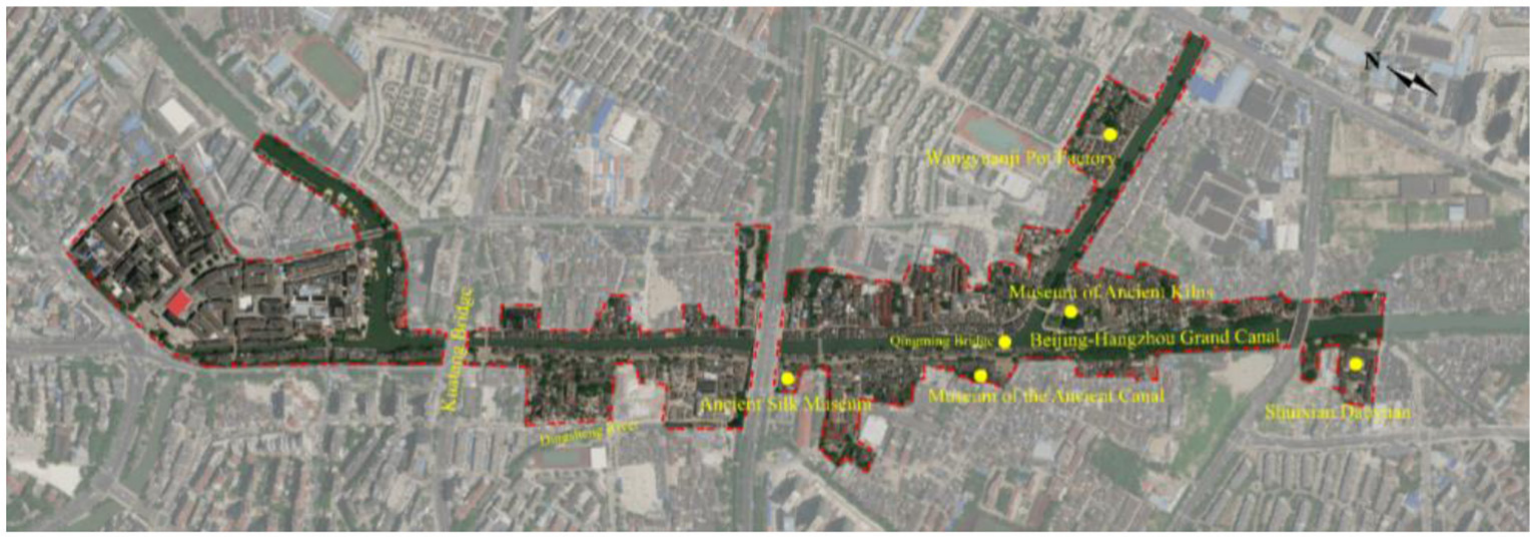
Satellite map of Qingming Bridge historical and cultural block.

Qingming Bridge Historical and Cultural District starts from Kuatang Bridge in the north, Shuixian Daoyuan in the south, the former site of Wangyuanji Pot Factory in the east, and along the Dingsheng River in the west. It covers an area of 0.44 square kilometers, and the core protected area covers an area of about 18.78 hectares (28). The block takes the ancient canal as the central axis and Qingming Bridge as the core. Nanchang Ancient Street and Nanxiatang Ancient Lane are arranged along the river, which is an important part of the North–South Waterway in China (Figure 2). The Qingming Bridge historical and cultural block was naturally formed in the historical process of north–south exchanges. The canal waterfront block, which was formed a long time ago, has not only become a place for the local residents′ daily life, living, and consumption, but also provides a short-term stay space for people traveling from the south to the north, and has now become a landmark cultural landscape in Wuxi. The historical and cultural value of Qingming Bridge Historical and Cultural Block is mainly manifested in the birthplace of Wu culture in China, the birthplace of modern Chinese national industry and commerce, and the original ecological features of Jiangnan people along the ancient canal (29).

In 2014, the Grand Canal of China was inscribed on the World Heritage List, and the 14 km river course of the “Wuxi Urban Section of the Jiangnan Canal” and the Qingming Bridge Historical and Cultural Block were listed as World Heritage River Courses and World Heritage Sites, respectively (30). For thousands of years, the Qingming Bridge Historical and Cultural Block was born and prospered by the river, continuing the historical context of the ancient charm of the canal. It offers a grand view, covering various types of resources, such as Jiangnan folk customs, national industry and commerce, water alleys, ancient architectural landscapes, and religions, forming the unique “Jiangnan historical and cultural landscape corridor.” It provides rich spiritual enjoyment for the people of Wuxi to seek “the taste of old Wuxi” (31, 32).

**Figure 3:**
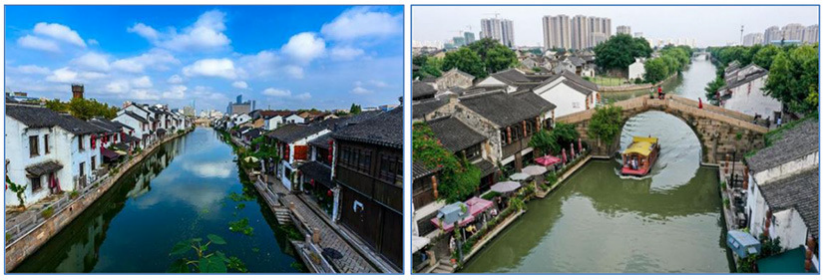
Street scene of Qingming Bridge historical and cultural block.

The block is known as “out-of-print canal, Jiangnan water alley,” and it is a unique historical and cultural block of the ancient canal in the south of the Yangtze River. There are a small number of ancient dwellings from the Ming and Qing Dynasties within the block. Most of the historical buildings were built in the late 19th and early 20th centuries, which have strong characteristics of Jiangnan canal family. There are courtyard-style, bamboo-tube-style, and free-standing river-pillow houses, as well as Chinese- and Western-style houses. One example is the perfect Shikumen merchant villa. At present, the business of the entire block is set up along the shoreline of the ancient canal along the Nanchang Street and Nanxiatang, mainly focusing on cultural and creative industries (Figure 3), with cultural and creative industries as the mainstay. On both sides of Dayao Road and Nanxiatang, there is still a large number of traditional Jiangnan dwellings. In recent years, with the integration and development of culture and tourism, it has also become the preferred typical case study for researchers in this field (33).

### Problem analysis

In terms of research theory and practice, the integration process of culture and tourism in the Qingming Bridge Historical and Cultural Block can be seen as a dividing point in 2014. Before this, the application for the status of World Heritage Site was the top priority. The content of the work was more inclined to the excavation, arrangement, and protection of the canal culture, and few authors were involved in the study of development and utilization of the heritage. After the successful application for a World Heritage Site in 2014, the research perspective of scholars underwent a major change because protection became the consensus of all, and the development and utilization of the cultural and sports heritage of the canal became the main object of discussion for more researchers.

From a theoretical perspective, the research literature on the protection, inheritance, and utilization of the cultural heritage of the canal is largely focused on three aspects. The first is research on the canal itself, focusing on the functional research of canal water conservancy, irrigation, and shipping. The second is research on the management system related to the canal, which mainly involves research on the management system at the national, provincial, and municipal levels. Most scholars believe that a systematic and unified management model should be established. The third is research on the development and utilization of cultural resources in the regions along the canal, and the directions of development and utilization mainly include tourism, recreation, and cultural creation.

From a practical perspective, it was found in the perception survey of tourists that tourists tend to pay too much attention to some unique and iconic landscapes. For example, most respondents were most impressed with Qingming Bridge, but knew little about another famous Wu Bridge landscape in the block. However, the whole section of the ancient canal that traverses Wuxi City is about six kilometers long, from Wu Bridge *via* Xishuidun, the South Gate, to Qingming Bridge. It indicates that the whole block still lacks effective overall marketing. In addition, through the analysis of tourists′ emotional attitudes, some negative comments were also found, mainly focusing on aspects such as “poor water quality” and “the scenic spot is not clean enough.” “Some tourists provided “no local characteristics,” “ordinary,” “that's it,” and other comments. It shows that tourists are paying more and more attention to the ecological environment protection and cultural theme characteristics of the block.

On the whole, with regard to the integrated development of culture and tourism in the Qingming Bridge historical and cultural block, theoretical and practical aspects often focus on the following issues: First, environmental issues, such as the water quality of the canal itself, environmental pollution and other ecological functions destruction, degradation and restoration; the second is social problems. There are still original residents living in the riverside houses, maintaining some original ecological scenes. However, due to disrepair and poor cultural relics protection, many famous hometowns and cultural classics have suffered to varying degrees. The third is the issue of economic development. The residents living on both sides of the river are mostly tenants, stranded elderly, and economically poor families. There is a significant contradiction between the preservation of traditional living space in the block and the expansion of economic space.

It can be said that no matter in terms of ecological environment, cultural protection or economic development, it shows a conservative and backward side, and is almost stuck in a dead end (34).

## Methodological framework

In view of the existing problems, it is necessary to find solutions from the theoretical and practical levels. This article adopts the pragmatic research method. As we generally believe that historical and cultural landscapes, represented by historic districts, play an important role in urban development and renewal, urban residents′ social participation, urban branding, and the citizens′ cultural identity. Identity and other aspects have a unique role (35, 36), which is highly consistent with the humanistic shift within the current urban economic development paradigm (37), and this is also consistent with the United Nations Sustainable Development Goals (SDGs) (38), the 2030 Agenda (39), the New Urban Agenda (40), and the New European Agenda (41), and other international and regional economic agendas are mutually confirmed. This has become important evidence for the realization of urban humanistic turn in different scales, such as on the global-, regional-, country-, and city-level scales. So this article adopts the pragmatic research method. The basic idea of this approach is to respect the emotion, will, development and needs of the individual case, and to provide solutions to real-world challenges by studying related issues from multiple perspectives (42). Since this method combines different analysis steps and logics, it can be well combined with the reality of the research object, which is very consistent with the current situation of economic globalization and diversification development, and becomes an appropriate method for current research problems (43). Based on this, the overall research idea of this article can be divided into two steps: first, through theoretical induction, text content analysis and other research methods, combined with field visits and investigations, explore and construct the overall framework of cultural tourism integration development; second, in the practical level, deductive reasoning and other methods are used, combined with the actual development of the research object area in recent years, to put forward strategic suggestions for the renewal and development of historical and cultural blocks.

### The humanistic shift in urban development

In the context of the return of urban humanism, to form an effective mode of harmonious interaction and repeated circulation of the elements of the historical block, the core foothold must be the real integration of cultural tourism, especially the realization of the humanistic shift in urban development. To a certain extent, the pursuit of the rationality of urban competition stems from the meaning of urban development and the rational interpretation of urban “ontology” (24). This so-called ontology, in the philosophical sense, refers to the ultimate existence, that is, the fundamental attributes, qualitative stipulations, and origins within things. The two major global ideological trends in the 20th century–scientism and humanism–essentially showed the macro-trend of human cultural development. Since humanism contains some reasonable elements from scientism, but from new heights and levels, a philosophical pattern dominated by the trend of humanism and the fusion of the humanistic and scientific spirit has been formed in contemporary society (44). The fundamental feature of modern human sciences is that the discussion of substance turns to the study of meaning, that is, from the concept of substance to the theory of meaning (45). “It is people, not technology, that must be the source of value; it is people, not the maximization of production, that should be the criterion for all planning” (46). Only by adhering to this principle, fully paying attention to all aspects of people's needs, and realizing the return of humanism, can urban public space realize its own value (47–50).

In fact, acknowledging the inclusiveness of humanism means that we have touched the cultural pulse that human culture itself is undergoing as a comprehensive re-examination of the scale of humanism; that is, modern ontology promotes the survival value of human beings (51). In a sense, the process of urban competition is the process of the city′s pursuit of “ontology,” and the deep-seated issues concerning the sustainable development of all cities are bound to unfold in the field of the city's ontology. If it is said that man is claimed to be a being that is constantly inquiring into himself–a being who must question and examine his condition at every moment of his existence (52). Then people′s exploration of coordinated urban competition and sustainable development will also become an overall revelation of and return to human existence and life activities.

### Regeneration framework

**Figure 4:**
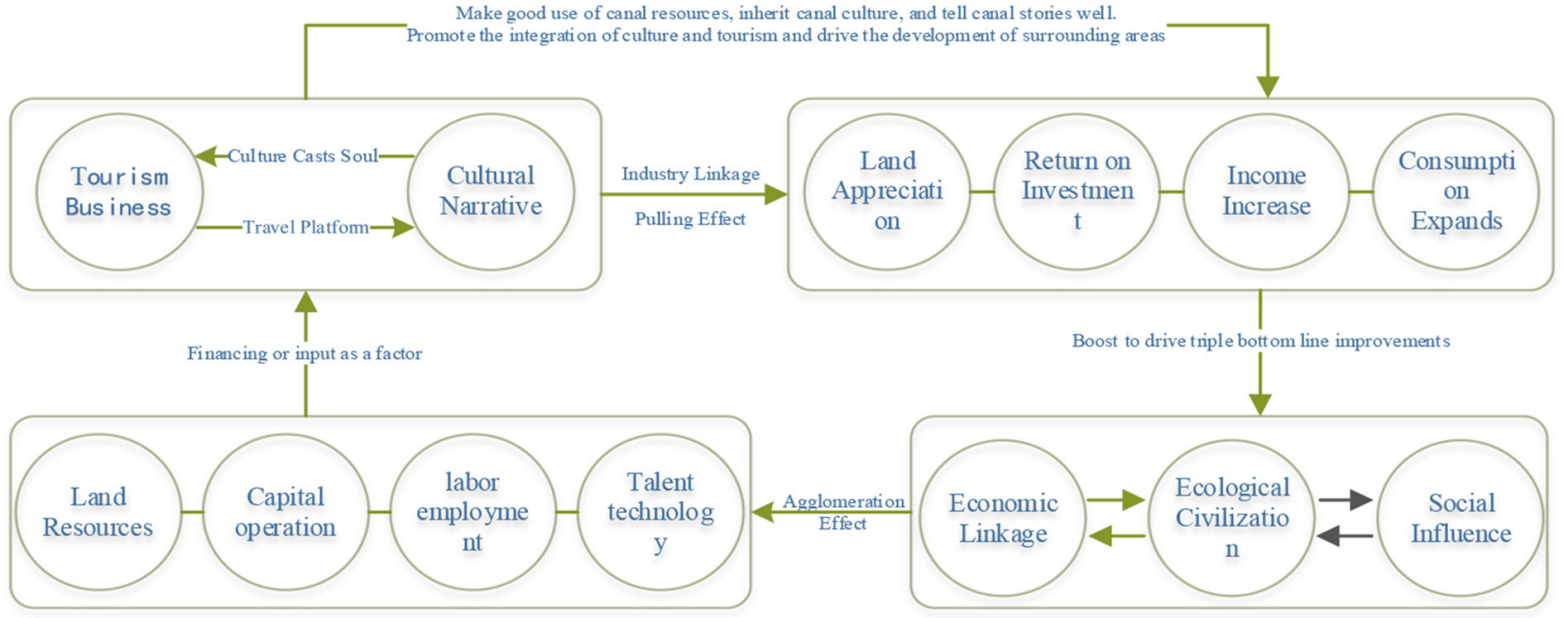
Framework for the renewal and development of historic districts.

From the previous analysis and the information summarized in Figure 1, COVID-19 has exerted a comprehensive impact on the development of urban blocks in terms of human resource supply, financial capital flow, commercial space utilization efficiency, and changes in consumption habits caused by anxiety. As a result, the return of economic production factors has dropped sharply, and the sustainable development capacity of the block has been greatly weakened. Therefore, it is urgent to regain consumer confidence and development confidence from the perspectives of public psychology and economic development. It can be divided into two levels in terms of its focus on people. One is materiality, which pays more attention to people's senses, including the external form of urban space, aesthetics and other sensory needs; the other is social and other spirituality, which pays more attention to people's inner feelings, such as possessive belonging, memory, social interaction, etc. (46). With the continuous improvement of people's needs, whether urban residents or foreign tourists, they will not only pay attention to the physical, economic, ecological and other tangible environments of historical and cultural blocks, but also have more general needs for the intangible environment at the historical and cultural level of the blocks. Therefore, on the basis of ensuring the construction of a good tangible environment, urban historical and cultural blocks must pay more attention to the protection and inheritance of spiritual and cultural aspects, and consider it in coordination with economic and social development, so as to jointly promote the development of various elements of the block into a virtuous circle state. Based on this, this article proposes that in the renewal and development of urban historical and cultural blocks, more attention should be paid to the rational arrangement of elements such as “two sectors,” “triple bottom lines,” and “four types of resources” (Figure 4), which fundamentally resolves the long-standing sharp conflict between the environment and development and ensures the sustainable development of the block.

Figure 4 reveals the updated development framework of historic districts in the context of the return of urban humanism. It acts on several main elements of economic development and can effectively resist the negative impact of COVID-19. First, the development of urban historical and cultural blocks must adhere to the bottom line thinking and respect the bottom line of the triple civilization development of “economy, ecology and society,” which is the basic guarantee for the sustainable development of blocks; Secondly, the development of any urban historical and cultural block is inseparable from the investment of four types of resource elements such as land, capital, labor, talent, and technology, the creation of hot consumption spaces, the gathering of cultural tourism talents, and the integration of commercial capital are indispensable development factors; Third, the tourism sector should form a positive interaction with the cultural sector, and the tourism industry should rely on the core cultural attractions, give full play to the advantages of the tourism industry chain, attract more market tourists to come to consume, and realize the drainage effect. The cultural sector should play an active and leading role, and on the basis of sorting out the canal heritage and cultural resources, extract the canal stories with local characteristics as the core attraction, so as to form an industrial linkage effect of cultural and tourism integration of “culture casts the soul, tourism sets up the stage,” and continuously obtains the return of the input elements. In this way, the overall improvement of the local economy, society and ecological environment will be further promoted, and the overall sustainable development of the block will be realized. To sum up, the sustainable development of historical and cultural blocks must “tell a compelling cultural story or create a beautiful atmosphere, follow the bottom line of the triple civilization development of “economy, ecology and society,” realize the integrated development of the two sectors of culture and tourism, and make the investment of “four types of resources” continue to get returns, and ultimately realize a virtuous circle of sustainable development.

## Regeneration paths of Qingming Bridge historic district

From the perspective of how COVID-19 affects urban economic development (Figure 1) and the renewal and development framework of urban historical blocks (Figure 4), the specific renewal paths of Qingming Bridge Historic District needs to be deployed at the following levels to form more normative development logic. The first is the spiritual and cultural aspect of the theme. The block needs to have its own characteristic theme. On the one hand, it distinguishes itself from other areas and forms its own unique development advantages. On the other hand, it must meet the strong needs of the public's spiritual and cultural life; Second, at the level of social and economic development, the condensing of cultural themes requires the leading role of the cultural sector, and also requires the tourism industry to play a role in marketing and tourist source drainage; Third, in terms of key development factors, it is necessary to bridge the negative impact of the epidemic, such as talent supply, capital withdrawal, insufficient space hot-spots, and insufficient consumption capacity, and achieve short-term economic recovery by reshaping these economic development factors; The fourth is to strictly follow the development experience of other canal regions in the world, and stick to the bottom line of economic, social and cultural development, so as to increase the capacity for sustainable development.

### Give full play to the core attraction role of cultural heritage resources

With humanities as the core and economy as the auxiliary, multi-line progress, cross-integration, a new development model for Qingming Bridge neighborhood under humanistic care can be established. The development model of block renewal is shown in Figure 4, which is mainly achieved through the utilization of local natural resources, community residents and space consumption, capital introduction, and talent accumulation. The two main sectors of culture and tourism should give full play to the role of the core attraction of cultural heritage resources, tell historical and humanistic stories well, attract people through tourism to improve the comprehensive benefits of the block, thereby driving the further value-added effects of the input elements. The value-added effect of the input factors is mainly reflected in the following aspects: first, it promotes the appreciation of the surrounding land; second, it promotes local employment, thereby increasing the income level of the residents, and third, it forms regional consumption hot-spots and allows consumption to increase. The long-term impact on local economic development will be reflected in the improvement of regional ecological benefits, social and economic benefits, and the resulting improvement in the quality of local economic growth factors. In this way, an economic feedback cycle of “resources–products–renewable resources” can be achieved.

Combining the local culture, tourism, and commercial resources of Wuxi, we can make in-depth attempts at the integrated development of culture, business, and tourism in the following aspects: First, by digging deep into the canal civilization, we can create a linear cultural and tourism gathering area along the river and form a natural physical space for a linear cultural and tourism gathering area, guiding the formation of a linear consumption space and finally achieving the spatial consumption effect. Second, we can activate the commercial port culture and create a planar cultural and tourism cluster based on urban space optimization. The commercial port culture was formed in Wuxi in ancient times and provides a preliminary agglomeration form in space. The focus of subsequent construction is to properly implant cultural creativity and activity themes in areas with excessive commercial development. Third, to improve the quality of tourism, there are many humanities-related resources in the block. Landscapes such as Nanchan Temple, Bodu Bridge, the ruins of kilns, and former residences of celebrities can be used to create slow walking trails, to connect various scenic spots with linear corridors, and to form linear or ribbon-shaped cultural tourism clusters. Landscapes and alleys form a staggered planar historical block landscape, driving the transformation and upgrading of urban tourist destinations and improving the overall quality of the block.

### Establish a comprehensive partnership between the two sectors of culture and tourism

The two most important sectors involve the cultural and the tourism industries. Since the two sectors serve different stakeholder groups, they have played different roles in society for a long time for different purposes. People in the tourism industry regard cultural resources as important raw materials for their tourism products to produce tourism-related products and economic wealth (53, 54), while people in the cultural management sector cherish these assets because of their intrinsic value and use them with very strict approaches to preserve these samples of cultural resources for the future, with the aim of serving the wider public interest, basically structured around public sector or non-profit organizations (55).

There may be various relationships between the two sectors of culture and tourism and the stakeholder groups they represent, such as comprehensive cooperation, working relationships, peaceful coexistence, minor dissatisfaction, initial conflict, and total conflict (56). Of course, according to the different roles played by the cultural sector and the tourism sector, this phenomenon can also be understood through the framework of a continuum. At one end of the continuum is the product in which the cultural sector occupies an absolutely dominant role, representing that the experience activities are designed with the goal of cultural resources or cultural heritage management as the center. At the other end of the continuum, it represents the dominant state of the tourism sector, and the tourism experience projects it develops tend to consider economic benefits more than the cultural connotation of the projects. Products that are in the middle of the continuum, culture and tourism are reflected in more partnerships. The more you go to the two ends of the continuum, the more contradictions and conflicts there are between the two sectors, because once the leading role of one sector is emphasized, the intervention of the other sector will be affected.

**Figure 5:**
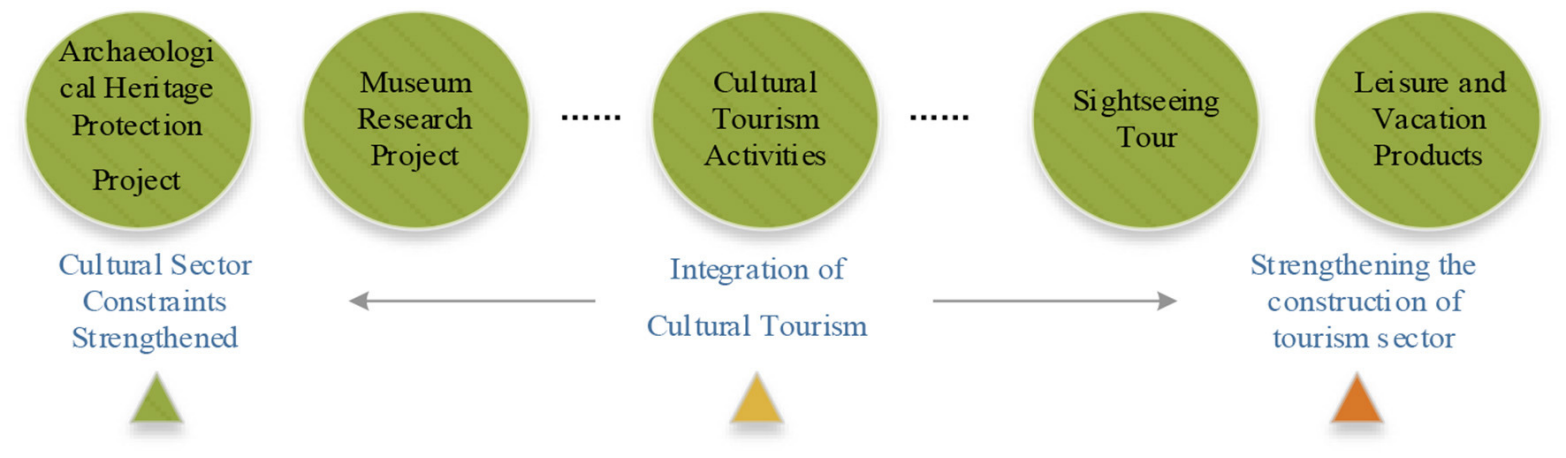
Schematic diagram of the product lineage of the integrated development of the two sectors of cultural tourism.

At present, the cultural sector of Qingming Bridge District and the tourism sector have carried out a series of measures, taking active actions in the aspects of cultural relic protection, economic development, and tourism prosperity, and have achieved certain results. However, it is obvious that the two sectors go their separate ways, and the cooperation results are less. In fact, if we look at the cooperation between the cultural and tourism sectors from the perspective of continuum, we can find more spaces for cooperation as shown in Figure 5 and develop a richer product spectrum. For example, when developing cultural protection projects such as archaeology and museums, the cultural sector can take a dominant position and play a central role, while the tourism sector can play a role in attracting tourists. When developing tourism activities such as sightseeing and leisure, the tourism sector should actively play the role of marketing and industrial development, while the cultural sector can create the cultural connotation and cultural theme of the project to reflect its degree of participation. In a word, there is no fundamental conflict of interest between the two sectors. All product development can be carried out with different degrees of cooperation between the two sectors of culture and tourism. The difference in product form or name only reflects the degree of participation of the two sectors. As long as the two sectors negotiate with each other in the process of different types of specific product cooperation, defining the division of labor and the degree of involvement of their respective roles can help to achieve a win–win situation to some extent.

### Pay more attention to the input of intangible cultural elements

The development momentum of the traditional tourism industry relies too much on the large-scale investment of material elements, such as those from nature and the environment, rather than advanced non-material elements, which once caused a series of secondary ecological civilization problems, such as the overuse of resources. The sustainable development of tourism has become an urgent problem to be solved (57). The integration and interaction process of the tourism and cultural industries is an effective way to move beyond the resource endowment-based development model. Cultural tourism promotes the transformation and upgrading of the traditional tourism industry to that of a modern service industry with the input of more non-material knowledge-based elements (58). By expanding the space for the development of the cultural industry, a new economic growth point is being formed (59). Among the many non-material knowledge-based elements, cultural elements can well-meet people's psychological needs of “seeking novelty, seeking differences, seeking happiness, and seeking knowledge,” so there is a huge space for breaking the traditional mold of tourism development.

At present, Qingming Bridge District is striving to expand its cultural resources, including mining the “four cultural context” resources, such as the canal culture, industrial and commercial culture, religious culture, and folk culture as shown in Table 1, and this has achieved certain results. In terms of inheriting the canal culture, well-known canal research experts are invited to determine the major historical events in the three-thousand-year history of the Grand Canal civilization, and to show the great civilizational history of the Grand Canal. In terms of highlighting the industrial and commercial culture, restoration of the former residences of the “Silk King” Xue Nanming and the “Electrical King” Zhu Dachun shows the brilliance of the folk kiln handicraft industry in the Ming and Qing Dynasties in an all-round and three-dimensional manner. In terms of promoting religious culture, the block cooperates with the religious sector to accelerate the transformation and upgrade of the Nanchan Temple Cultural Mall, realizing the integrated construction of the pagoda and courtyard in the development of folk customs. In terms of culture, the block uses the restored residential buildings in Nanchang Street and Nanxiatang as the carriers to create the “most Wuxi” time-honored homestay area. It can be said that Qingming Bridge District has paid attention to the inheritance and protection of culture during the development process. However, there is still a slight lag in capital operation, labor employment, talent technology. It relies mostly on the support of the government sectors, which fails to give full play to the advantages of various resources.

**Table 1:** Summary and development update of intangible cultural elements.

**Resource elements**	**Summary of cultural elements**	**Formation of consumption space**
Canal heritage	Determining the major historical events of the canal and showing the history of the canal civilization.	Canal linear consumption space.
Industrial and commercial remains	Highlighting two typical characters: the “Silk Industry King” and the “Electrical King.”	Xue Nanming's former residence and Zhu Dachun's former residence.
Places of worship	Cooperating with the religious and cultural sectors to carry out the integrated construction of the tower and courtyard and carrying forward the religious culture.	Nanchan Temple Cultural Mall renovation and upgrade.
Residential buildings	Creating the “most Wuxi” time-honored home-stay area, reflecting the local people's customs and characteristics.	Restoration of Nanchang Street and Nanxiatang residential buildings.

### Stick to the triple bottom line of economy, society, and environment

The “triple bottom line” highlights that development integration must operate in accordance with all three of the economy, society, and environment. The integration between sectors and industries needs to be based on a broad social consensus, that is, development and integration issues should be coordinated with a sustainable concept which is more in line with social responsibility. Specifically, the integration of culture and tourism requires comprehensive consideration of the happiness of the community, the satisfaction of tourists, the wishes of the cultural resources sector, and so on. For example, not all cultural resources have tourism potential, and not all cultural resources with tourism potential can receive many tourists. For some cultural resources, based on their tourism potential or tourism value, it is optimal to achieve their economic value; for other cultural resources, perhaps protection is the most appropriate (60–62).

**Figure 6:**
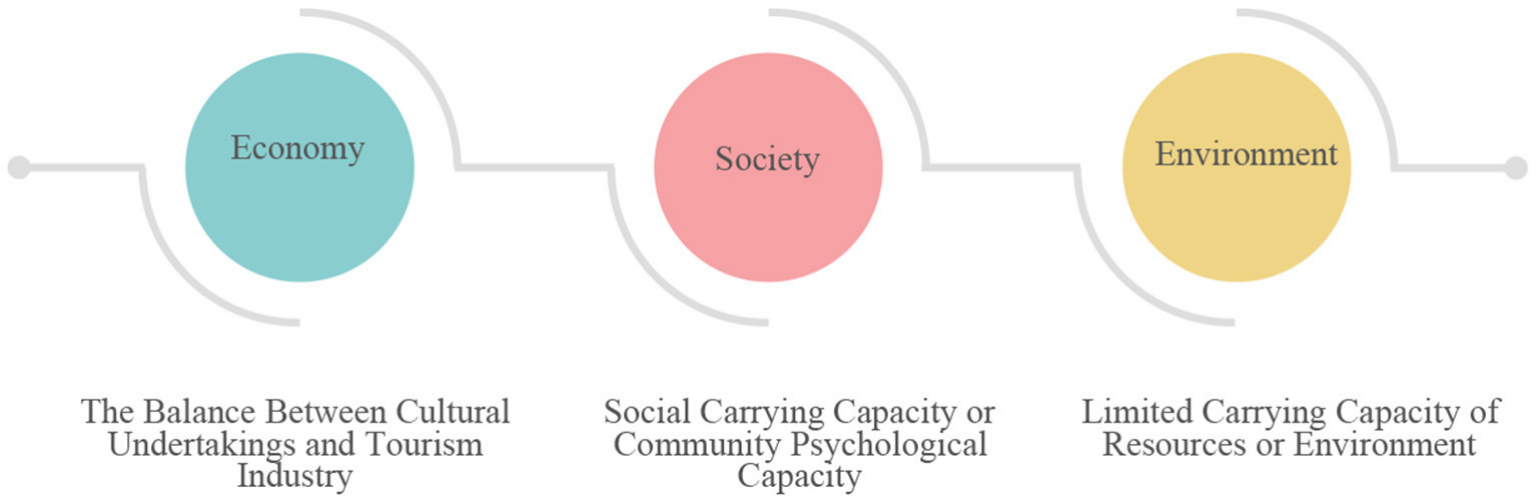
The “triple bottom line” in the integration development of culture and tourism.

Compared with the past-history of Qingming Bridge District, the construction of the current district has been greatly improved. At present, guided by the creation of a high-quality urban leisure tourism resort with international standards, the block actively learns from the latest development concepts of advanced regions in the world. With the general principles of “promoting development,” “creating characteristics,” and “increasing vitality,” it focuses on the cultural industry and the tourism industry. With the joint efforts of the two sectors of culture and tourism, effective work has been carried out in both the determination of cultural and the utilization of tourism resources, and good results have been achieved. However, the relationship between the economy, society, and environment is itself intricate. At present, most of the residents of Qingming Bridge have moved out, and the number of commercial complexes has increased, especially those within the hotel and catering industry, which will definitely damage the environment. In addition, how to further systematize the protection of historical and cultural points not only concerns selling tickets in a circle but is also closely related to the improvement of Wuxi society and the refinement of the classification of tourism levels. In the context of the triple bottom line, how to minimize conflicts and give full play to complementary advantages is the development direction of Qingming Bridge District as shown in Figure 6.

## Conclusion and discussion

### Conclusion

Many cities pursue the growth of material factors too much in the process of development, they pay too much attention to the increase of GDP value, the growth of economic income, and the increase of the number and scale of enterprises, while ignoring the spiritual and cultural needs of the public. The COVID-19 epidemic has also fully exposed the problems in urban development. Many cities have experienced public mental health problems, conflicts and disputes between communities and tourists, and conflicts of interest between different economic sectors. These phenomena and problems are actually about spiritual or cultural aspects.

The integrated development of the culture and tourism industries in Qingming Bridge Historical and Cultural Block has become an effective way to expand development space, promote structural adjustment, and enhance regional competitiveness (63, 64). In the context of the return of urban humanism, the article has constructed an analytical framework for the integrated development of culture and tourism in historical and cultural blocks, and analyzed the path and practice of Wuxi Qingming Bridge Historical and Cultural Blocks to achieve sustainable development. Based on the historical and cultural blocks of Qingming Bridge, this article analyse the integration and development of its culture and tourism. It is believed that the sustainable development of Qingming Bridge Historical and Cultural Block must be based on the comprehensive investment of “four types of resources,” such as culture, land, capital, and labor, and rely on the cooperation of the “two sectors” of culture and tourism to achieve the sustainable development of the regional ecology, economy, and society. This article moves beyond the previous technical route of analyzing the integration of culture and tourism from the perspective of the tourism industry and integrated non-industrial cultural factors into the development process of cultural tourism; that is, we have used the perspective of cultural tourists, cultural motivation, and the depth of experience as the basis for destination selection, provides a new perspective for the study of cultural tourism integration.

## Discussion

The Jiangnan Canal heritage on which the block is based is also a linear, flowing, and cross-administrative area that can be considered as an organic whole (65, 66). With the cooperation of the cities along the route, the canal heritage culture in the historical dimension will be deeply excavated. Based on the premise of protection, more emphasis should be placed on following the principles of “overall regional planning, combination of protection and utilization, adaption of measures to local conditions, and highlighting characteristics,” which can promote exchanges and cooperation in matters such as regional brand building, cross-border cooperation, resource integration, and experience sharing. This will help to achieve cultural development within the process of regional integration and provide a high degree of unified ecological, social, and economic benefits (67).

In the future, the cultural heritage of the Jiangnan Canal can be further used as a breakthrough point, and the canal can be transported or used as a water source (function-related). Areas that are spatially adjacent to the canal (spatial production) and related to important people or things in the development and evolution of the canal (social historical and cultural dimension) reshape the cultural heritage elements of the canal in three aspects and balance the sustainable relationship between cultural heritage protection and inheritance, as well as cultural innovation development and utilization. However, the research of this article also has some shortcomings. The article mainly analyzes the ideas of the integrated development of culture and tourism industry from the macro level, and lacks systematic comparative research and reference to canal cities in other countries in the world. These contents will be paid attention to in the follow-up research.

## Data availability statement

The original contributions presented in the study are included in the article/supplementary material, further inquiries can be directed to the corresponding author.

## Author contributions

Conceptualization and writing—original draft preparation: FL and MG. Methodology and supervision: MG. Validation: MG and LL. Formal analysis: FL and YL. Investigation: FL and YP. Resources and funding acquisition: FL. Writing—review and editing: MG and YL. Project administration: YP. All the authors have read and agreed to the published version of the manuscript.

## Funding

This study was funded by a General Project of Humanities and Social Sciences Research of Ministry of Education: Research on the Poetics of Kazuo Ishiguro's Novels in the context of Trans-boundary (Grant No. 22YJA752006) and the General Project of Philosophy and Social Sciences Research in Jiangsu Universities Research on the Ecological Reconstruction Corpus of Language Landscape in Southern Jiangsu Province (Grant No. 2019SJA0827).

## Conflict of interest

The authors declare that the research was conducted in the absence of any commercial or financial relationships that could be construed as a potential conflict of interest.

## Publisher's note

All claims expressed in this article are solely those of the authors and do not necessarily represent those of their affiliated organizations, or those of the publisher, the editors and the reviewers. Any product that may be evaluated in this article, or claim that may be made by its manufacturer, is not guaranteed or endorsed by the publisher.
